# Assessing Urinary Metabolomics in Giant Pandas Using Chromatography/Mass Spectrometry: Pregnancy-Related Changes in the Metabolome

**DOI:** 10.3389/fendo.2020.00215

**Published:** 2020-04-16

**Authors:** Maosheng Cao, Chunjin Li, Yuliang Liu, Kailai Cai, Lu Chen, Chenfeng Yuan, Zijiao Zhao, Boqi Zhang, Rong Hou, Xu Zhou

**Affiliations:** ^1^College of Animal Sciences, Jilin University, Changchun, China; ^2^Chengdu Research Base of Giant Panda Breeding, Sichuan Key Laboratory of Conservation Biology for Endangered Wildlife, Sichuan Academy of Giant Panda, Chengdu, China

**Keywords:** giant pandas, metabolomics, tryptophan, choline, kynurenic acid

## Abstract

Giant pandas represent one of the most endangered species worldwide, and their reproductive capacity is extremely low. They have a relatively long gestational period, mainly because embryo implantation is delayed. Giant panda cubs comprise only a small proportion of the mother's body weight, making it difficult to determine whether a giant panda is pregnant. Timely determination of pregnancy contributes to the efficient breeding and management of giant pandas. Meanwhile, metabolomics studies the metabolic composition of biological samples, which can reflect metabolic functions in cells, tissues, and organisms. This work explored the urinary metabolites of giant pandas during pregnancy. A sample of 8 female pandas was selected. Differences in metabolite levels in giant panda urine samples were analyzed via ultra-high-performance liquid chromatography/mass spectrometry comparing pregnancy to anoestrus. Pattern recognition techniques, including partial least squares-discriminant analysis and orthogonal partial least squares-discriminant analysis, were used to analyze multiple parameters of the data. Compared with the results during anoestrus, multivariate statistical analysis of results obtained from the same pandas being pregnant identified 16 differential metabolites in the positive-ion mode and 43 differential metabolites in the negative-ion mode. The levels of tryptophan, choline, kynurenic acid, uric acid, indole-3-acetaldehyde, taurine, and betaine were higher in samples during pregnancy, whereas those of xanthurenic acid and S-adenosylhomocysteine were lower. Amino acid metabolism, lipid metabolism, and organic acid production differed significantly between anoestrus and pregnancy. Our results provide new insights into metabolic changes in the urine of giant pandas during pregnancy, and the differential levels of metabolites in urine provide a basis for determining pregnancy in giant pandas. Understanding these metabolic changes could be helpful for managing pregnant pandas to provide proper nutrients to their fetuses.

## Introduction

Giant pandas (*Ailuropoda melanoleuca*) are rare endangered mammals with high social and scientific value. The prerequisite for ensuring better protection of endangered species is a good understanding of their reproductive physiological characteristics. Giant pandas were confirmed to have embryo diapause in 2009 (1); that is, during pregnancy, the embryo floats in the womb and stops developing until it attaches to the womb a few months later. Embryo implantation is delayed in giant pandas, resulting in a long gestational period. Although the giant panda has a longer pregnancy, the newborn cub is underdeveloped, weighing only 90–130 g (2). Conversely, the average adult giant panda weighs 75–160 kg (3). The main reason for this phenomenon is that giant pandas adapt to unpredictable food sources (4). Giant pandas experience embryo diapause during pregnancy, and some giant pandas experience false pregnancy. Pseudopregnant pandas will exhibit the same physiological and behavioral changes as pregnant pandas. Thus, it is difficult to identify pregnancy in giant pandas based on changes in progesterone levels (5). At present, research on captive giant pandas has focused on the detection of estrus and mating (6, 7), as well as infant care and development (2, 8). However, there are sparse reports on the physiological changes of pregnant pandas. Untimely pregnancy testing in giant pandas in captivity interferes with their raising and breeding, further inhibiting efficient breeding and management. Therefore, the physiological characteristics of giant pandas during pregnancy must be better understood to effectively protect these animals.

Metabolomics is the study of the composition of metabolites in biological samples. These metabolites reflect the metabolic functions of cells, tissues, and organisms. Moreover, metabolomics has an advantage over genomics, transcriptomics, and proteomics because metabolites are generated by ongoing biological processes in the body, and they therefore more accurately and directly reflect phenotypic changes. In addition, it is increasingly being used to identify potential biomarkers to help identify and prevent diseases (9). Among the various biological matrices used in metabolomics, urine is characterized by non-invasive collection methods and the presence of abundant metabolites that reflect all biochemical pathways in the body (10). Metabolomics involves comparing metabolic concentrations between different samples, and this innovative technique is used extensively to search for biomarkers. The metabolome can be analyzed using various techniques (gas chromatography-mass spectrometry [MS], liquid chromatography [LC]-MS, and nuclear magnetic resonance). Ultra performance LC-MS (UPLC-MS) combines high-throughput processing, high sensitivity, and high resolution to obtain more accurate, reliable, and comprehensive data compared with these other techniques. UPLC-MS can identify and semi-quantify 100 s to perhaps about 1,000 of metabolites simultaneously and generate a large amount of MS data. Pattern recognition techniques, such as principal component analysis (PCA), partial least squares-discriminant analysis (PLS-DA), and orthogonal PLS-DA (OPLS-DA), are used to analyze data pertaining to multiple parameters after large amounts of data have been obtained (11–13). Finally, these MS data can be interpreted by combining these pattern recognition analyses with bioinformatics analysis. Bioinformatics analysis primarily involves the use of XCMS, MZmine 2 and MS-DIAL (14, 15) software for substance detection and metaX software (16) for substance quantification and differential substance screening. Using these software programs, a large amount of data can be screened to identify highly relevant metabolites in different physiological states, analyze the relevant metabolic pathways of these differential metabolites, identify potential molecular mechanisms and their biological significance, and find additional biomarkers for different physiological states. Analyzing the relevant metabolic pathways of differential metabolites can help identify the potential mechanisms and provide information about their biological significance, as well as facilitate the identification of biomarkers that indicate different physiological states. In recent years, metabolomics has been increasingly applied to the study of pregnancy and pregnancy-related diseases (17), as well as to studying the role of small-molecule metabolites in pregnancy and pregnancy-related diseases from a holistic perspective (18). These studies can be performed using maternal blood, urine, amniotic fluid, and placental tissue. To date, urine has been used as a biological matrix for metabolomic analysis to study the relationship between gestational diabetes mellitus and metabolic disorders (19) to identify urinary biomarkers of aberrant fatty acid and carbohydrate metabolism in early pregnancies complicated by gestational diabetes (20), identify potential biomarkers of fetal malformation and premature delivery in the second trimester (21), and study urinary metabolic changes during and after pregnancy (22). Urinary metabolomics plays an important role in the study of gestational stages in human pregnancy, but to date, the metabolomics of pregnant giant pandas has not been analyzed. One of the main difficulties of panda breeding is the low survival rate of newborns. The twin rate of giant pandas is 48.1%, and the average number of births is 1.48. However, there are often large differences in the birth weights of twin cubs, meaning that the smaller cubs are underdeveloped, making it difficult for them to survive after birth (2, 23). After childbirth, giant pandas support their infants on their bodies for the first 1–2 weeks of life. They will forego feeding and drinking to focus on lactation and thermoregulation (2, 24). In mammals, fetuses require a significant amount of maternal nutrients at the last stage of development to ensure their proper growth. At this point in pregnancy, maternal metabolism changes dramatically (25). Therefore, metabolomic analysis of urine from pregnant giant pandas is a promising means of identifying metabolic biomarkers of late pregnancy in giant pandas. If pregnancy is confirmed, then interventions, such as dietary and lifestyle changes, can be made to effectively promote fetal growth and development.

In this work, metabolites in urine from pregnant giant pandas were explored via UPLC-MS, and changes in amino acid and lipid metabolism were identified. Our results provide important new insights into the metabolites produced by giant pandas during pregnancy, and they may help identify metabolic biomarkers that can be used to detect changes in fetal nutrient requirements during late pregnancy, which could then be targeted to promote efficient reproduction in these endangered animals.

## Materials and Methods

### Study Animals and Urinary Samples

Giant pandas live in the Giant Panda Breeding Research Base in Chengdu, Sichuan Province, People's Republic of China. In this study, eight captive, sexually mature female giant pandas were investigated. Of the eight giant pandas, four participated in the breeding program of 2017 (all four had successful births). Another four giant pandas participated in the breeding program in 2018 (all four had successful births). All eight animals were inseminated via artificial insemination or natural mating (Table S1).

Urine samples (~3 mL each) were collected from eight pandas during anoestrus (20–30 days before mating) and pregnancy (100–110 days after mating). Urine samples were collected from a cement floor using clean glass droppers with rubber heads. The urine samples were immediately transferred to clean 15-mL centrifuge tubes and labeled with the animal identification number and date of collection. All samples were stored at −80°C until analysis. The collected samples were thawed on ice, and metabolites were extracted from 20 μL of each sample using 120 μL of precooled 50% methanol buffer. The extraction mixture was then vortexed for 1 min, incubated at room temperature for 10 min, and stored overnight at −20°C. The next day, the mixture was centrifuged at 4000 × *g* for 20 min, and then the supernatant was transferred to 96-well plates. The samples were stored at −80°C prior to the LC-MS analysis. Pooled quality control (QC) samples were also prepared by combining 10 μL of each extraction mixture (26).

### Urinary Progestogen Assay

A monoclonal antibody progestogen enzyme immunoassay [CL425; C. Munro (27)] was used to quantify the progesterone concentration in urinary samples. Creatinine (Cr) is used as an indicator of the progesterone concentration to adjust for variability in urine dilution (28), and the values are expressed as mass/mg Cr (Table S1).

### LC-MS Analysis Conditions

All samples were analyzed using a TripleTOF 5600 Plus high-resolution tandem mass spectrometer (SCIEX, Warrington, UK). Chromatographic separation was performed using an UPLC system (SCIEX). An ACQUITY UPLC T3 column (100 mm × 2.1 mm, 1.8 μm, Waters, UK) was used for the reversed-phase separation. The injection volume for each sample was 4 μL per run. The mobile phase consisted of solvent A (water and 0.1% formic acid) and solvent B (acetonitrile and 0.1% formic acid). The gradient elution conditions were as follows: 5% solvent B for 0–0.5 min; 5–100% solvent B for 0.5–7 min; 100% solvent B for 7–8 min; 100–5% solvent B for 8–8.1 min; and 5% solvent B for 8.1–10 min. The column temperature was maintained at 35°C. The flow rate was 0.4 mL/min.

The TripleTOF 5600 Plus system was used to detect metabolites eluted from the column. The quadrupole time-of-flight (Q-TOF) mass spectrometer was operated in both positive- and negative-ion modes. The curtain gas pressure was set at 30 PSI, the ion source gas 1 and gas 2 pressure was set at 60 PSI, and the interface heater temperature was 650°C. For the positive-ion mode, the ion spray floating voltage was set at 5,000 V, and for the negative-ion mode, the ion spray floating voltage was set at −4500 V. The MS data were acquired in the IDA mode. The TOF mass range was 60–1200 Da. Survey scans were acquired every 150 ms, and as many as 12 product ion scans were collected if the threshold of 100 counts/s was exceeded with a 1+ charge state. The total cycle time was fixed at 0.56 s. Four time bins were summed for each scan at a pulse frequency of 11 kHz by monitoring the 40-GHz multichannel TDC detector with four-anode/channel detection. Dynamic exclusion was set for 4 s. During the acquisition, the mass accuracy was calibrated every 20 samples. To evaluate the stability of the LC-MS during the entire acquisition period, a QC sample (created by pooling all of the samples) was analyzed after every 10 experimental samples.

### Metabolomics Data Processing

Before the group data analysis was performed, the group datasets were normalized. Data normalization was performed on all samples using the probabilistic quotient normalization algorithm. Then, QC-robust spline batch correction was performed using QC samples. The acquired LC-MS raw data were analyzed using XCMS software (SCIEX), including peak picking, peak grouping, retention time correction, second peak grouping, and annotation of isotopes. Raw LC-MS data files were converted into the mzXML format and then processed using the XCMS, CAMERA, and metaX (16) toolboxes included in R software. Each ion was identified by combining retention time and m/z data. The intensity of each peak was recorded, and a three-dimensional matrix containing arbitrarily assigned peak indices (retention time–m/z pairs), sample names (observations), and ion intensity information (variables) was generated. This primary MS information was then matched to secondary information from our in-house database. First-order MS information was used for identification, and second-order information was used for matching to the in-house standard database. The Kyoto Encyclopedia of Genes and Genomes (KEGG, http://www.kegg.com/) and Human Metabolome Database (HMDB, http://www.hmdb.ca) were used to annotate the metabolites by matching the exact molecular mass data (m/z) of the samples to those from the database to identify the physicochemical properties and biological functions of the metabolites. The peak intensity data were further preprocessed using metaX (16). Features that were detected in <50% of the QC samples or 80% of the biological samples were removed, and values for missing peaks were extrapolated using the k-nearest neighbor algorithm to further improve the data quality. PCA was performed to detect outliers and evaluate batch effects using the pre-processed dataset. QC-based robust LOESS signal correction was fitted to the QC data with respect to the order of injection to minimize signal intensity drift over time. In addition, the relative standard deviations of the metabolic features were calculated across all QC samples, and those with standard deviations >30% were removed. The analysis methods included PCA and PLS-DA. MetaX software was used to quantify differential metabolites and differential metabolite screenings. Supervised PLS-DA was conducted using metaX to identify variables that differed between groups. The variable importance in projection (VIP) was calculated, and a VIP cutoff of 1.0 was used to select important features (VIP ≥ 1; ratio ≥ 2 or ratio ≤ 1/2; *q* ≤ 0.05).

## Results

### Untargeted Metabolic Profiling of Urine During Anoestrus and Pregnancy

**Figure 1:**
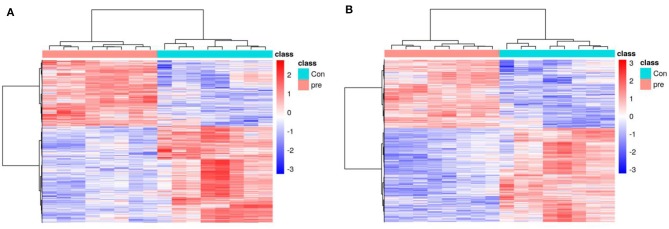
A heatmap of the metabolites identified in the urine of pregnancy and non-pregnancy giant pandas. **(A)** Positive ions, **(B)** Negative ions.

To explore the metabolic changes in urine during pregnancy in giant pandas, an untargeted metabolomics analysis was performed, which identified 7,831 annotated metabolites from 12,451 positive-ion features (Table S2) and 5,744 annotated metabolites from 12,664 negative-ion features (Table S3). The total ion chromatograms of positive-ion mode, negative-ion mode, and QC samples were analyzed using UPLC/Q-TOF MS, and the chromatographic separation spectra exhibited good overlapping (Figure S1). In addition, high-resolution mass spectra were analyzed using the TripleTOF 5600 Plus instrument, including the m/z width and retention time width (Figures S2, S3), with the results meeting the standards. Figures 1A,B presents an overview of the metabolic changes in urine during anoestrus and pregnancy based on positive- or negative-ion analysis. The results illustrated that urinary metabolites varied greatly between pregnant and non-pregnant giant pandas.

Annotations revealed that many different types of metabolites were present in the urine. In the positive-ion mode, the 20 largest metabolic categories were arachidonic acid metabolism (69 metabolites), 2-oxocarboxylic acid metabolism (36 metabolites), tyrosine metabolism (36 metabolites), serotonergic signaling (33 metabolites), and steroid hormone biosynthesis (32 metabolites) (Figure S4). In the negative-ion mode, the 20 largest metabolic categories were arachidonic acid metabolism (35 metabolites), steroid hormone biosynthesis (28 metabolites), neomycin, kanamycin, and gentamicin biosynthesis (21 metabolites), 2-oxocarboxylic acid metabolism (19 metabolites), and amino sugar and nucleotide sugar metabolism (17 metabolites) (Figure S5).

### Multivariate Statistical Analysis

**Figure 2:**
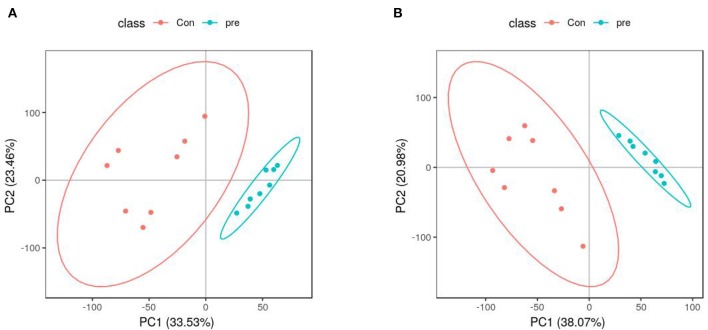
Principal component analysis score plots of metabolites identified in urine during anoestrus and pregnancy. **(A)** Positive ions, **(B)** Negative ions.

**Figure 3:**
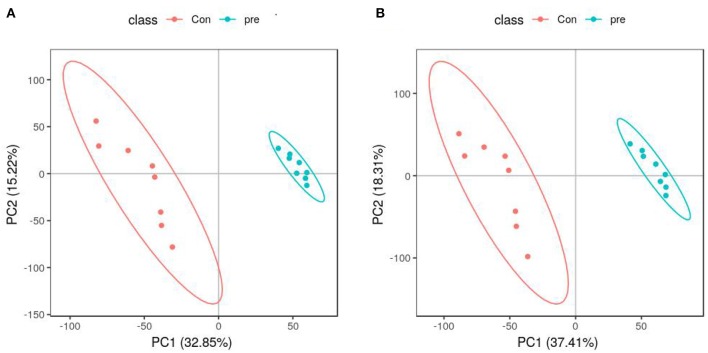
Partial least squares-discriminant analysis score plots of metabolites identified in urine during anoestrus and pregnancy. **(A)** Positive ions, **(B)** Negative ions.

PCA was used to determine the sample separation and aggregation between the pregnant and non-pregnant pandas. Each point on the PCA score graph represents a single sample. Aggregation of points indicates that the observed variables are highly similar, and discrete points represent significant differences (VIP ≥ 1; ratio ≥ 2 or ratio ≤ 1/2; *q* ≤ 0.05) in the observed variables. In the positive-ion mode, the PCA scores illustrated that PC1 and PC2 were responsible for 33.53 and 23.46% of the variation, respectively (Figure 2A). In the negative-ion mode, the PCA scores revealed that PC1 and PC2 were responsible for 38.07 and 20.98% of the variation, respectively (Figure 2B). The results demonstrated that urine from anoestrus and pregnancy had different metabolic characteristics. This indicated a clear separation between giant panda urine metabolites during anoestrus and pregnancy. PCA is mainly used to observe separation between groups in an experimental model, but it cannot identify specific changes between groups. Therefore, PLS-DA, which is a supervised discriminant profiling statistical method, was used to identify more specific differences between the groups. Higher values for PLS-DA model parameters (R2 and Q2) denote greater reliability for the PLS-DA model. R2 of the PLS-DA model in the positive-ion mode was 0.986, and Q2 was 0.943 (Figure 3A). R2 of the PLS-DA model in the negative-ion mode was 0.985, and Q2 was 0.953 (Figure 3B), indicating that both R2 and Q2 were high. According to the PLS-DA model parameters, this model was credible for interpreting the differences; therefore, we used these data for subsequent analyses.

**Figure 4:**
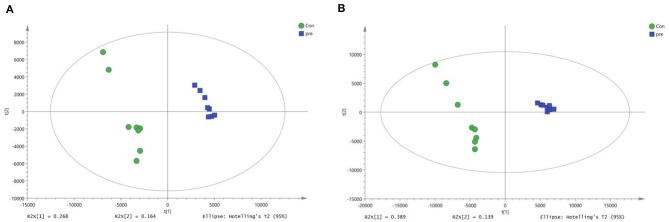
Orthogonal partial least squares-discriminant analysis score plots of metabolites identified in urine during anoestrus and pregnancy. **(A)** Positive ions, **(B)** Negative ions.

Next, OPLS-DA was used to analyze the metabolites in urine during anoestrus and pregnancy. In the positive-ion mode, the OPLS-DA parameters were as follows: R2X = 0.433, R2Y = 0.991, and Q2 = 0.922 (Figure 4A). In the negative-ion mode, the OPLS-DA parameters were as follows: R2X = 0.529, R2Y = 0.993, and Q2 = 0.971 (Figure 4B). The intercept of the OPLS-DA model did not reach the overfitting threshold (R2Y > 0.4, Q2Y > 0.05). The OPLS-DA score plot demonstrated a clearer separation of the urine between anoestrus and pregnancy samples. The Q2 values all exceeded 0.4, indicating that the current OPLS-DA model was more reliable and that consistent modeling and predictability were achieved.

### Potential Biomarkers and Metabolic Pathways for Metabolite Analysis

**Figure 5:**
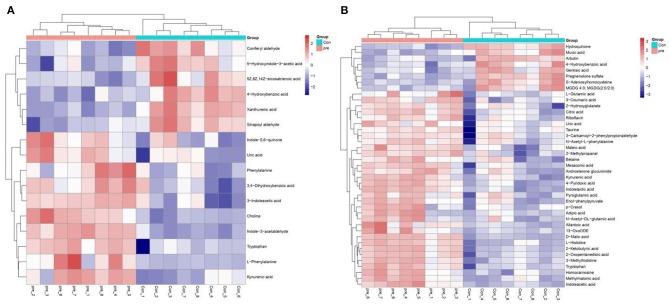
Heatmaps of metabolites present at significantly different levels in urine during anoestrus and pregnancy. **(A)** Positive ions, **(B)** Negative ions. Red indicates an increase, blue indicates a decrease, rows indicate different metabolites, and columns indicate different samples.

Next, we subjected the metabolomics data to univariate analysis of fold changes and T statistical testing to perform Benjamini–Hochberg correction and obtain the q-value. This was combined with multivariate statistical analysis of the VIP obtained via PLS-DA to screen for differential metabolites. Differential ions were defined as follows: VIP ≥ 1; ratio ≥ 2 or ratio ≤ 1/2; *q* ≤ 0.05. Based on these criteria, a comprehensive statistical analysis was performed to compare urine from pregnant and non-pregnant giant pandas. In the positive-ion mode, 8,702 characteristic ions were detected, of which 896 were present at higher levels and 1,307 were present at lower levels in during pregnancy (Figure S6). In the negative-ion mode, 9,152 characteristic ions were detected, of which 1,210 were present at higher levels and 1,662 were present at lower levels in during pregnancy (Figure S6). LC-MS data analysis was used to analyze the secondary metabolites of different substances. Secondary differential metabolite ions were defined according to the aforementioned criteria. Exact mass data (m/z) from the KEGG and HMDB databases were used to annotate 59 differential metabolites. Sixteen differential metabolites were found in the positive-ion mode, and 43 differential metabolites were found in the negative-ion mode. For unsupervised clustering, the significantly different metabolites were used to construct heatmaps (Tables S4, S5). A heatmap was used to define the metabolites with different levels in urine taken during anoestrus and pregnancy. Consistent with the OPLS-DA results, both the positive- and negative-ion modes revealed significant aggregation (Figure 5). These results indicated significant changes in the expression of molecules associated with amino acid metabolism, lipid metabolism, and organic acid production during pregnancy. The changes in amino acid metabolism occurred in tryptophan (Trp), tyrosine, and methionine metabolism. Regarding Trp metabolism, Trp, indoleacetic acid, l-glutamic acid, and kynurenic acid were present at significantly higher levels in pregnant pandas, whereas xanthurenic acid and S-adenosylhomocysteine (SAH) were present at significantly lower levels. Concerning tyrosine metabolism, l-glutamic acid and indole-5,6-quinone were present at significantly higher levels in the pregnant group, whereas SAH was present at significantly lower levels. Regarding methionine metabolism, choline, betaine, and 2-ketobutyric acid were present at significantly higher levels in the pregnant group, whereas SAH was present at significantly lower levels. These data indicate that l-glutamic acid and SAH are involved in multiple amino acid metabolic pathways. Concerning lipid metabolism, androsterone glucuronide, adipic acid, mesaconic acid, and d-malic acid were present at significantly higher levels in pregnant pandas, whereas pregnenolone sulfate was present at significantly lower levels. These metabolic changes during late gestation may reflect changes in fetal nutrients needs, as well as the metabolic status of the mother.

## Discussion

Urine is characterized by abundant metabolite levels that reflect all biochemical pathways within the body (10). In this study, a MS-based approach to urine metabolomics and biomarker discovery was used to examine urinary metabolism via UPLC Q-TOF/MS analysis. Urine from 8 giant pandas during anoestrus and subqequent pregnancy was tested to identify changes in metabolite levels associated with different pathways involved in pregnancy. This study identified 59 differential metabolites via UPLC Q-TOF/MS analysis. In the positive-ion mode, 16 differential metabolites were found; notably, Trp, kynurenic acid, 3-indoleacetic acid, and choline were present at higher levels in pregnant pandas, whereas 4-hydroxybenzoic acid, coniferyl aldehyde, and xanthurenic acid were present at lower levels. In the negative-ion mode, 43 differential metabolites were found. Notably, 2-ketobutyric acid, 2-oxopentanedioic acid, l-histidine, and indoleacetic acid were present at higher levels in pregnant pandas, whereas pregnenolone sulfate and SAH were present at lower levels. These differential metabolites represent changes in various metabolic processes, such as amino acid and lipid metabolism, as well as organic acid content. Choline is an essential nutrient for animals, and its metabolites phosphatidylcholine and sphingomyelin are the main lipid components of the cell membrane (29). Choline is involved in lipid metabolism, brain development, and fetal development, and it is a key component of the cell membrane (30). Studies have found that choline levels are increased in maternal plasma during pregnancy, and they are supplied to the fetus via one-way transfer through the placenta (31). Maternal choline supply to the fetus plays an important role in fetal brain development, membrane biosynthesis, and neurotransmission. In rat models, prenatal choline supplementation protected memory in adults and temporal and spatial memory in the offspring (32). Although no studies have examined this in pandas, we hypothesize that this is one of the explanations of the increase in choline levels in giant panda urine during late pregnancy. Choline supplementation during pregnancy is also important in humans. Infant brain development can be improved by maternal supplementation with phosphatidylcholine, a choline metabolite, during pregnancy (33). When the body has high choline content, choline enters the mitochondria, in which it is metabolized to betaine aldehyde by choline oxidase, and betaine aldehyde is further oxidized to betaine by betaine aldehyde dehydrogenase. When the body lacks choline, the betaine content will decrease (34). Betaine is mainly produced by choline in the body, and plasma betaine levels are positively correlated with the choline concentration (35). Homocysteine is strongly and negatively correlated with plasma betaine (35). Homocysteine is a sulfur-containing amino acid that can be converted to methionine via the remethylation pathway. Serum homocysteine levels are significantly lower in pregnant women than in non-pregnant women (36). Therefore, homocysteine can be used as a marker of pregnancy. SAH can be hydrolyzed to homocysteine by SAH hydrolase. Prior research found that SAH accumulation indirectly induces homocysteine toxicity. In the present study, we found that the urine of pandas during pregnancy contained 6-fold more choline than during anoestrus, as well as higher betaine levels. This indicates that choline and betaine also play important roles in giant pandas during pregnancy. SAH levels were significantly lower when giant pandas were pregnant compared to anoestrus. Because homocysteine is a metabolite of SAH, we speculated that homocysteine levels may also decrease during pregnancy in giant pandas. This speculation is consistent with previous studies indicating that serum homocysteine levels are significantly lower in women of childbearing age than in non-pregnant women (36). Although we did not analyze homocysteine levels in this study, our results indicate that choline plays a key role in maintaining pregnancy in giant pandas. Urinary SAH levels could therefore be used as a biomarker of pregnancy in pandas.

**Figure 6:**
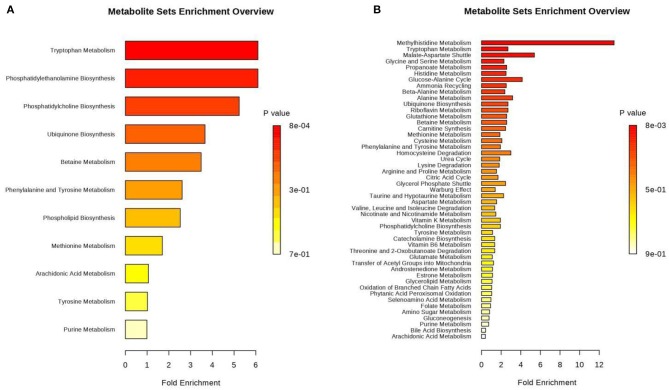
Histogram of differential metabolites annotated by comparison to the Kyoto Encyclopedia of Genes and Genomes (KEGG) database. Differential metabolites were classified by KEGG pathway enrichment and significance analysis. Fold enrichment is presented as the ratio of the number of metabolites assigned to the modified pathway by enrichment analysis to the theoretical number of metabolites assigned to the modified pathway by random distribution. The degree of enrichment is indicated by different colors in the histogram according to the *p*-value. **(A)** Positive ions, **(B)** Negative ions.

We respectively performed pathway analysis of differential positive and negative ions. The results from both the positive- and negative-ion modes indicated that Trp metabolism plays an important role in pregnancy (Figure 6). Trp is an essential amino acid that plays an important role in protein synthesis, and it is a precursor of many biologically active substances, including 5-hydroxytryptamine (5-HT), kynurenine, and kynurenic acid. 5-HT is an important neurotransmitter in the central nervous system (37) that can be further converted to melatonin. Research illustrated that Trp-rich foods or intravenous Trp can promote melatonin secretion (38). Therefore, Trp is also a precursor of melatonin, making it a regulator of circadian rhythms. Trp is metabolized by multiple metabolic pathways, and most free Trp is metabolized via the kynurenine pathway. Trp is degraded to kynurenine via indoleamine 2,3-dioxygenase (IDO) and Trp 2,3-dioxygenase (TDO). Trp plays an important role in maintaining pregnancy in humans. Fetal demand for protein increases continuously during pregnancy. Trp metabolites include 5-hydroxychromone, kynurenine, and kynurenic acid, which have different effects on signal transduction, immunosuppression, and neuronal protection. Studies have revealed that the placenta is the most abundant source of IDO enzymes (39). During pregnancy in humans, Trp levels in maternal plasma decrease because IDO in the placenta promotes local Trp metabolism (40), which increases kynurenine levels in the placenta, thereby inhibiting maternal T-cell proliferation and protecting the fetus from the maternal immune system (41). Although maternal plasma Trp levels decrease during human pregnancy, free (i.e., not bound to albumin, the form immediately available for tissue uptake) and total Trp levels are increased during pregnancy in rats (42–44). During the first 12 days of pregnancy in rats, free and total Trp levels increase in the mother because TDO activity is inhibited in the liver (45). Inhibition of TDO activity affects Trp metabolism. Levels of progesterone and estradiol, which inhibit TDO, increase in early pregnancy (45, 46). Therefore, free Trp levels are higher in pregnant mothers than in non-pregnant women. In the present study, urinary Trp levels during pregnancy were 4.9-fold higher than those observed during anoestrus. Thus, Trp levels increase and play an important role during pregnancy in giant pandas. We speculate that Trp levels increase during pregnancy because TDO activity in the liver is inhibited and Trp metabolism is reduced, thereby promoting fetal development (Figure S7). In the present study, we also detected an increase in the levels of kynurenic acid, which is part of the Trp metabolism pathway. We hypothesized that placental IDO promotes Trp metabolism in the umbilical cord, thus promoting kynurenine synthesis. Studies indicated that exogenous kynurenine supplementation promotes an increase in kynurenic acid levels in both the fetus and mother. However, exogenous kynurenic acid supplementation did not increase kynurenic acid levels in either the fetus or the mother (47). Therefore, we speculate that the elevated kynurenic acid levels in the urine of pregnant giant pandas may be attributable to the increased kynurenine content in the cord blood, and the presence of kynurenine promotes kynurenic acid production via the activity of kynurenine transaminase. Kynurenine is metabolized to 3-hydroxykynurenine (3-HA) via kynurenine mono-oxygenase (KMO), and xanthurenic acid is a metabolite of 3-HA. In our study, xanthurenic acid levels were reduced in the urine during pregancy compared to anoestrus, possibly because the upregulation of estrogen and progesterone production inhibits KMO expression during pregnancy (48).

## Conclusion

Metabolomic profiling of urine from giant pandas sampled during anoestrus and pregnancy was performed using UPLC Q-TOF/MS. Fifty-nine metabolites were present at different levels in the urine when samples from pregnancy were compared to anoestrus samples. These metabolites included amino acid and lipid metabolites. To our knowledge, this is the first study to investigate metabolic changes in pregnant giant pandas. Our study provides new insights into urinary metabolite changes in giant pandas during pregnancy and provides a basis for detecting pregnancy in giant pandas and identifying biomarkers that indicate fetal nutrient requirements during late gestation.

## Data Availability Statement

The datasets generated for this study can be found in: https://www.ebi.ac.uk/metabolights/MTBLS1574 (49).

## Ethics Statement

The animal study was reviewed and approved by Chengdu Research Base of Giant Panda Breeding.

## Author Contributions

XZ and CL designed the experiments, reviewed, and revised all subsequent versions of the manuscript. MC performed the experiments and prepared the first draft of the manuscript. All authors contributed in the literature search, discussion of the published evidence, read and approved the final manuscript, made substantial direct intellectual contributions to the work, and approved its submission for publication.

### Conflict of Interest

The authors declare that the research was conducted in the absence of any commercial or financial relationships that could be construed as a potential conflict of interest.
