# Supplementary material for: Assessing Urinary Metabolomics in Giant Pandas Using Chromatography/Mass Spectrometry: Pregnancy-Related Changes in the Metabolome
Source: Front Endocrinol (Lausanne). 2020 Apr 16;11:215. doi: 10.3389/fendo.2020.00215 (PMC7176934; doi:10.3389/fendo.2020.00215)
Supplement: Supplementary file 6 [file Image_1.PDF]

Supplementary Figures

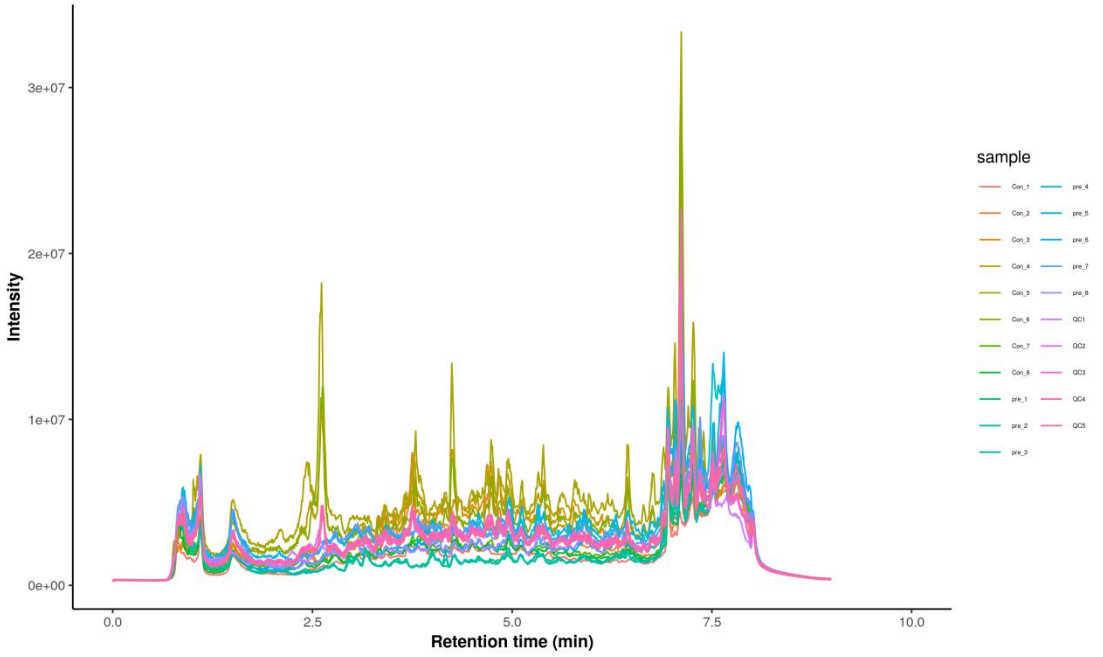

Figure S1. The total ion chromatograms of all the samples.

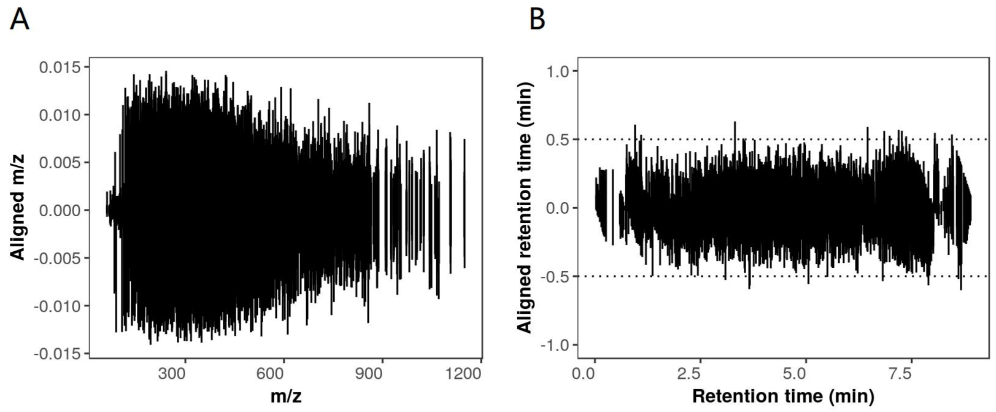

Figure S2. The m/z width A and retention time width B of our MS data under positive ion mode.

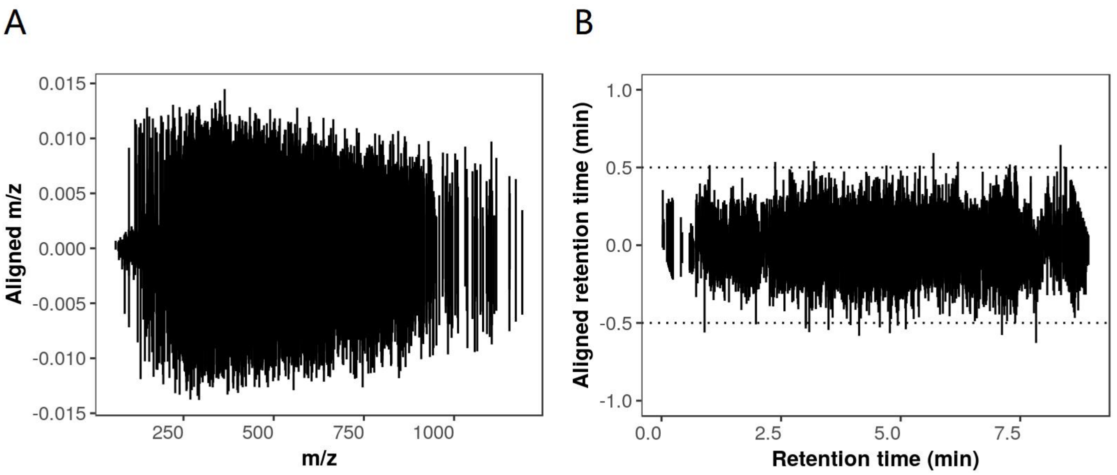

Figure S3. The m/z width A and retention time width B of our MS data under negative ion mode.

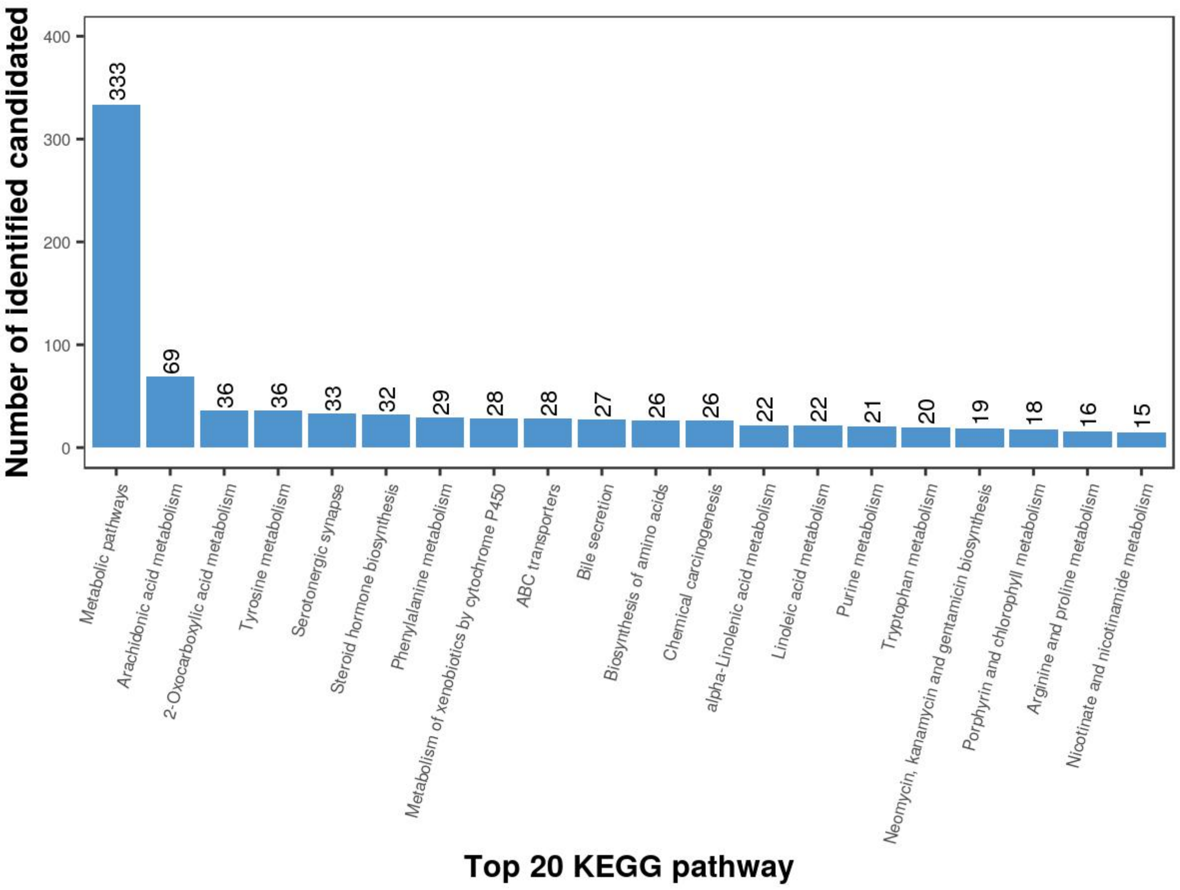

Figure S4. The top 20 largest metabolic categories of all identified metabolites under positive ion mode.

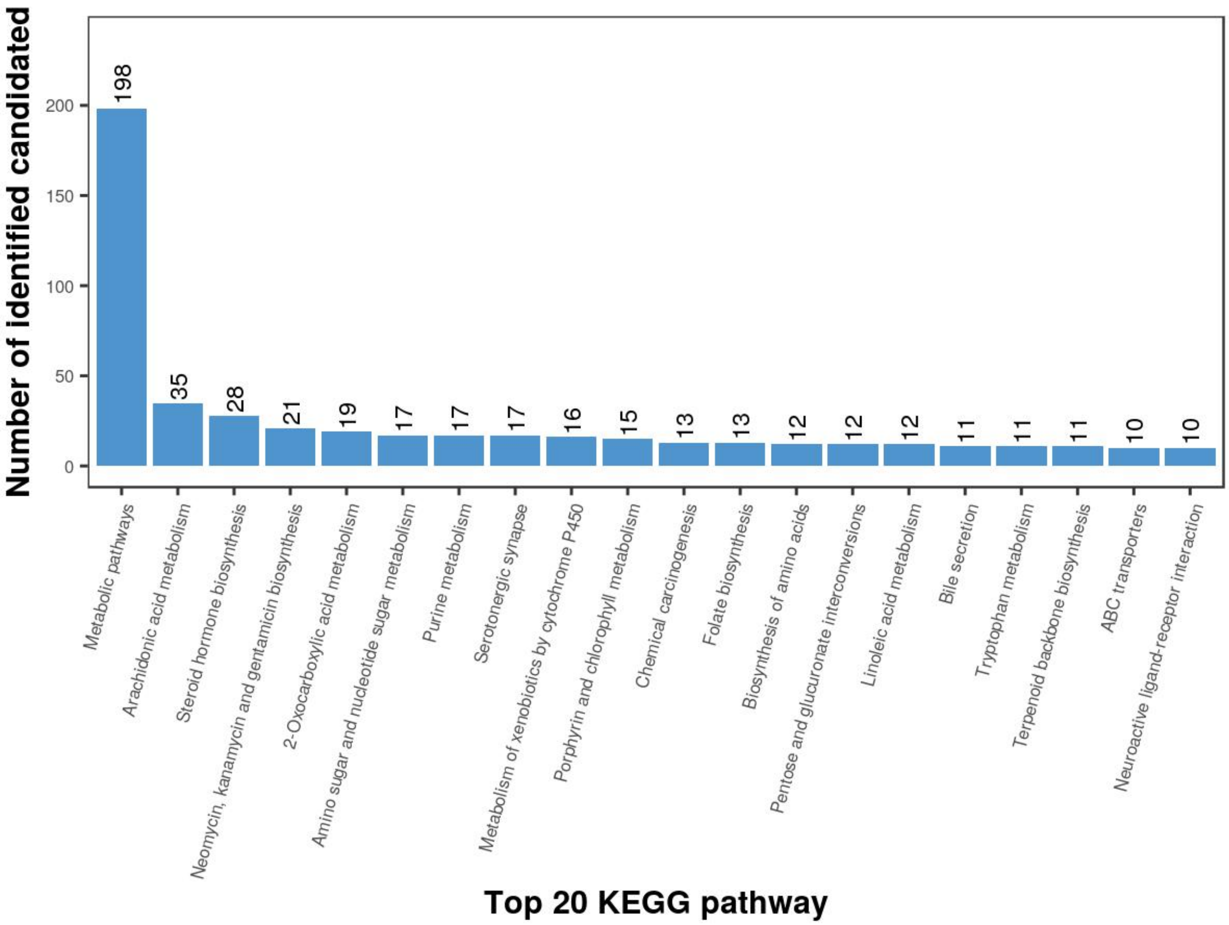

Figure S5. The top 20 largest metabolic categories of all identified metabolites under negative ion mode.

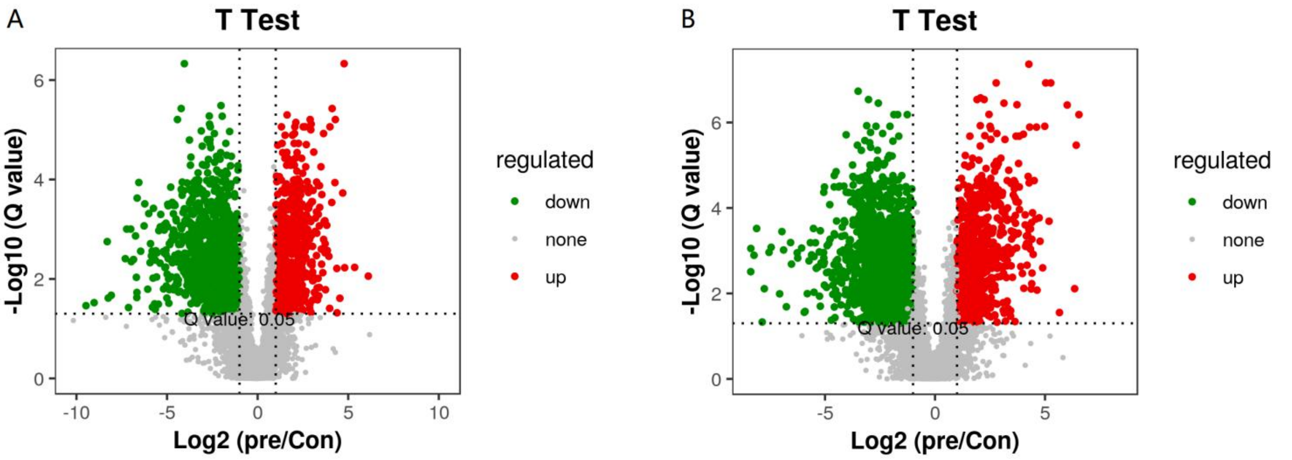

Figure S6. (A) Differential ion speed in positive ion mode, (B) Differential ion speed in negative ion mode.

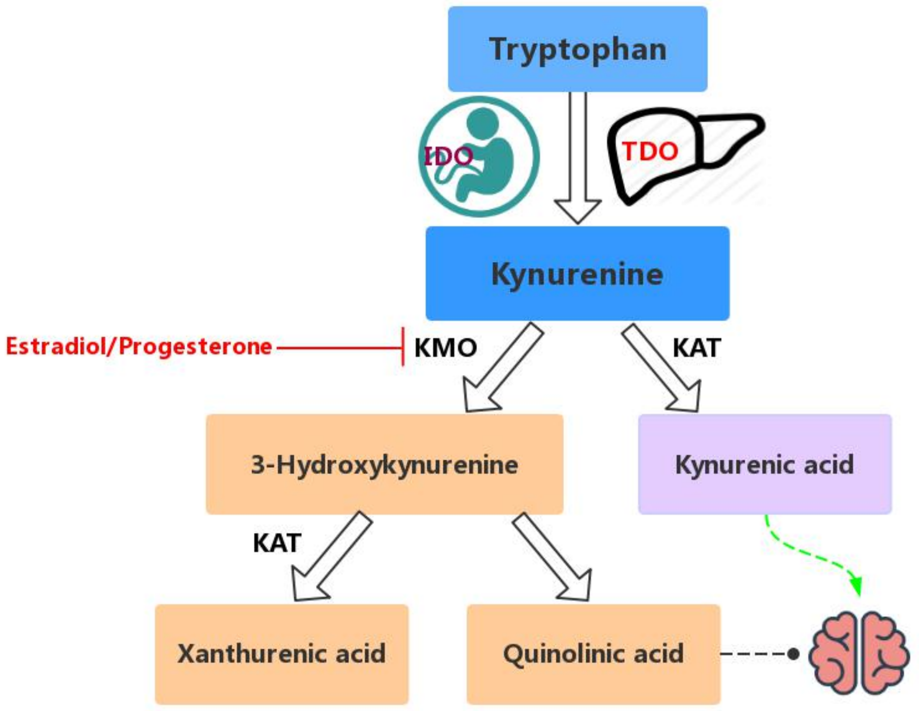

Figure S7. Metabolic map of tryptophan through the kynurenine pathway, The first step in this pathway is the conversion of tryptophan to kynurenine under the action of indoleamine 2-3 deoxygenase (IDO) and tryptophan-2,3-dioxygenase (TDO). The second step of this pathway is to convert Kynurenine to 3-hydroxykynurenine (3HK) and kynurenic acid under the action of kynurenine monooxygenase (KMO) and kynurenine aminotransferase enzymes (KAT). KMO can be inhibited by estradiol or progesterone (red arrow) (Reddy and Bethea, 2005). 3HK can be

metabolized into several neuroactive compounds, including quinolinic acid (QA), Xanthurenic acid. In the case of depression, KynA may have neuroprotective effects, while 3HK and QA may be neurotoxic (Bay-Richter et al., 2015; Savitz et al., 2015).

References

Bay-Richter, C., Linderholm, K.R., Lim, C.K., et al. (2015). A role for inflammatory metabolites as modulators of the glutamate N-methyl-D-aspartate receptor in depression and suicidality. Brain, behavior, and immunity 43, 110-117. doi:10.1016/j.bbi.2014.07.012

Reddy, A.P., and Bethea, C.L. (2005). Preliminary array analysis reveals novel genes regulated by ovarian steroids in the monkey raphe region. Psychopharmacology 180(1), 125-140. doi:10.1007/s00213-005-2154-1

Savitz, J., Drevets, W.C., Smith, C.M., et al. (2015). Putative neuroprotective and neurotoxic kynurenine pathway metabolites are associated with hippocampal and amygdalar volumes in subjects with major depressive disorder. Neuropsychopharmacology 40(2), 463-471. doi:10.1038/npp.2014.194
